# Pharmacological hypotheses: Is acetaminophen selective in its cyclooxygenase inhibition?

**DOI:** 10.1002/prp2.835

**Published:** 2021-07-18

**Authors:** Christopher J. Esh, Bryna C. R. Chrismas, Alexis R. Mauger, Lee Taylor

**Affiliations:** ^1^ Aspetar—Qatar Orthopaedic and Sports Medicine Hospital Research and Scientific Support Aspire Zone Doha Qatar; ^2^ School of Sport, Exercise and Health Sciences Loughborough University Loughborough UK; ^3^ Department of Physical Education College of Education Qatar University Doha Qatar; ^4^ Endurance Research Group School of Sport and Exercise Sciences University of Kent Chatham Maritime UK; ^5^ School of Sport, Exercise and Rehabilitation Faculty of Health University of Technology Sydney (UTS) Sydney Australia; ^6^ Human Performance Research Centre, Faculty of Health, University of Technology Sydney (UTS) Sydney Australia

**Keywords:** acetaminophen, arachidonic acid, cyclooxygenase, mechanism of action

## Abstract

The precise mechanistic action of acetaminophen (ACT; paracetamol) remains debated. ACT’s analgesic and antipyretic actions are attributed to cyclooxygenase (COX) inhibition preventing prostaglandin (PG) synthesis. Two COX isoforms (COX1/2) share 60% sequence structure, yet their functions vary. COX variants have been sequenced among various mammalian species including humans. A COX1 splice variant (often termed COX3) is purported by some as the elusive target of ACT’s mechanism of action. Yet a physiologically functional COX3 isoform has not been sequenced in humans, refuting these claims. ACT may selectively inhibit COX2, with evidence of a 4.4‐fold greater COX2 inhibition than COX1. However, this is markedly lower than other available selective COX2 inhibitors (up to 433‐fold) and tempered by proof of potent COX1 inhibition within intact cells when peroxide tone is low. COX isoform inhibition by ACT may depend on subtle in vivo physiological variations specific to ACT. In vivo ACT efficacy is reliant on intact cells and low peroxide tone while the arachidonic acid concentration state can dictate the COX isoform preferred for PG synthesis. ACT is an effective antipyretic (COX2 preference for PG synthesis) and can reduce afebrile core temperature (likely COX1 preference for PG synthesis). Thus, we suggest with specificity to human in vivo physiology that ACT: (i) does not act on a third COX isoform; (ii) is not selective in its COX inhibition; and (iii) inhibition of COX isoforms are determined by subtle and nuanced physiological variations. Robust research designs are required in humans to objectively confirm these hypotheses.

## Graphical abstract

Despite decades of use and research the precise mechanism of action of acetaminophen (ACT) is still not fully understood. It has been proposed that ACT targets a cyclooxygenase (COX) 1 splice variant (often termed COX3) or selectively inhibits COX2 however, no physiologically functional COX3 has been sequenced in humans and there is evidence of potent COX1 inhibition by ACT. The hypothesis of this paper is that ACT mediated COX inhibition is determined by subtle and nuanced physiological variations.

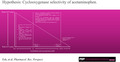

**Abbreviations**
ACT: acetaminophen
AM404: N‐acylphenolamine
CB1: cannabinoid 1
COX: cyclooxygenase
LPS: lipopolysaccharide
NSAIDS: non‐steroidal anti‐inflammatory drug
PG: prostaglandin
PGG2: prostaglandin G2
PGH2: prostaglandin H2
5‐HT: serotonin
TRPV1: transient receptor potential vanilloid 1
TXB2: thromboxane B2

## 1 INTRODUCTION

Acetaminophen (ACT; also known as paracetamol) is an effective and safe analgesic/antipyretic drug, used as early as 1893.^1^ Erroneously, phenacetin was preferred to ACT at this time due to a perceived greater safety profile; however, it was found to have a role in analgesic nephropathy.^2^ In 1949 it was established that the therapeutic efficacy of phenacetin was due to its metabolite ACT,^3^ with phenacetin use subsequently discontinued in the United Kingdom (1980) and United States (1983).^4^ Thereafter, ACT use increased markedly, currently used by 60 million people per week in the United States.^5^ ACT has similar functions (i.e., analgesic/antipyretic) to non‐steroidal anti‐inflammatory drug (NSAIDs). Despite their use since the late 1800s, the mechanism of action of NSAIDs [inhibition of prostaglandin (PG) synthesis] was not elucidated until 1971.^6^ More precisely, NSAIDs exert their action on the cyclooxygenase (COX) enzyme.^6^ Initially, due to ACT’s weak anti‐inflammatory and antiplatelet action it was not thought to inhibit COX.^6^ However, ACT was subsequently found to inhibit COX in the brain.^7^

The COX enzyme is the catalyst for the rate‐limiting steps that synthesize PG’s.^1^, ^8^ COX oxidizes arachidonic acid resulting in the production of prostaglandin G2 (PGG2 before peroxidization to prostaglandin H2 (PGH2), this compound is metabolized via precise enzymatic activities to produce the desired PG.^8^ Central to defining the mechanism of action of ACT (and NSAIDs) was the determination of a second COX isoform in 1991.^9^, ^10^, ^11^ These COX isoforms (COX1 and COX2) share 60% structural sequence identity,^12^ yet their expression and function can vary. COX1 has been attributed “housekeeping” functions and is constitutively expressed in most tissues, maintaining homeostasis (e.g., gastric cytoprotection and hemostasis^12^, ^13^), while, COX2 is inducible, expressed in various pathophysiological states (e.g., inflammation^12^, ^13^). However, the assigning and general superficial acceptance of such isoform specific functions, likely, oversimplifies these highly complex isoforms and is sometimes inaccurate.^12^ Indeed, there may be some constitutive COX2 expression/function^14^, ^15^, ^16^, ^17^; attributing any in vivo molecule/biomarker a specific function in complex hosts such as humans must be done so with caution, particularly when attempting to determine the mechanism(s) of drug action.^18^, ^19^ Throughout this paper, the use of COX refers to the combination of COX1 and COX2 and the individual isoforms will be named specifically when referring to their individual action.

ACT’s mechanistic actions are not fully elucidated and remain under investigation.^20^, ^21^ After the second COX isoform was discovered,^9^, ^10^, ^11^ several further COX variants have been sequenced, in humans and other mammals^22^, ^23^; most discussed of these is COX3.^24^ Some claim this as the elusive target of ACT’s action^20^, ^24^, ^25^, ^26^ while others refute the COX3 hypothesis.^22^, ^27^, ^28^, ^29^ Parallel to the COX3 hypothesis are debates of whether ACT is selective in its COX1 and/or COX2 inhibition, or not.^30^, ^31^ Table 1 provides an overview of research that has investigated the in vivo mechanism of action of ACT and its proposed target. This paper will discuss the evidence for the hypotheses that ACT, with specificity to human in vivo physiology: (i) does not act on a third COX isoform; (ii) is not selective in its COX inhibition; and (iii) inhibition of COX isoforms are determined by subtle and nuanced biological variations.

**TABLE 1:** Key research investigating the in vivo mechanism of action of ACT and its proposed target

Study	Species	Proposed target of ACT
Chandrasekharan et al.^24^	Canine (cerebral cortex) Insect (cells)	COX3
Ayoub et al.^25^	Mouse	COX3
Ayoub et al.^26^	Mouse	COX3
Ayoub and Flower^20^	Mouse	COX3 or other COX1 gene derived protein
Li et al.^27^	Mouse	COX2 (febrile antipyretic) Unclear afebrile hypothermic action
Hinz et al.^30^	Human	COX2
Lee et al.^48^	Human	COX2

## 2 COX3: ACETAMINOPHENS TARGET COX ENZYME?

**FIGURE 1:**
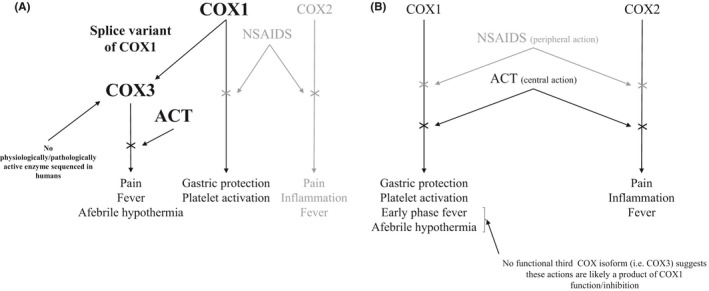
Schematic of hypothesis 1: ACT does not inhibit a third cyclooxygenase (COX3) isoform. Panel (A) The proposed COX3 mechanism of action of ACT. A splice variant of COX1 named COX3^24^ has exhibited physiological and pathological function in mice, canine, and insect models.^20^, ^24^, ^25^, ^26^ This is not the case in other mammals [humans/rats etc^22^]. Panel (B) The more traditional schematic of the mechanism of action of ACT. Both ACT and NSAIDS inhibit COX1/2. NSAIDS generally exhibit a more peripheral action on COX hence a high anti‐inflammatory/antiplatelet action,^26^, ^37^ whereas ACT has a more central mechanism of action and displays only analgesic and antipyretic function.^31^ On the assumption that there is no functional third COX isoform, the afebrile hypothermia and early phase febrile actions are likely a result of COX1 activity in humans. Abbreviations; ACT, acetaminophen; COX, cyclooxygenase; NSAID, non‐steroidal anti‐inflammatory drugs

ACT’s mechanistic action is distinct from traditional NSAIDs,^1^ with weak anti‐inflammatory^31^ and/or antiplatelet action^32^ alongside superior gastrointestinal safety.^33^ Intuitively, ACT’s COX1/2 inhibitory mechanism of action^31^, ^32^ has been questioned. Born out of this was the plausibility of the existence of an unidentified COX isoform being highly sensitive to ACT inhibition.^34^ Figure 1 displays a visual representation of the traditional and proposed (i.e., COX3) ACT/COX inhibition mechanisms. COX3, an alternatively spliced messenger ribonucleic acid (mRNA) variant of COX1, was found in the canine cerebral cortex.^24^ The fact that this enzyme is not genetically distinct and its gene mRNA is identical to COX1 except for the retention of intron 1,^32^ the naming of this enzyme as COX3 is refuted by some.^22^, ^29^ However, for the purposes of this paper COX3 will be used. The catalytic properties of the three COX enzymes (COX1‐3) were assessed through PGE2 concentration post exogenous arachidonic acid administration in insect cells.^24^ COX2 demonstrated the greatest catalytic activity [COX3 exhibited ~4% of the activity of COX2^24^]. Subsequently, COX 1–3 sensitivity to inhibition via ACT was determined; the COX3 enzyme had the lowest IC_50_ value of the three COX enzymes (COX3: 64 µmol·L; COX1: 133 µmol·L; COX2: 5887 µmol·L) in the presence of 5 µmol·L arachidonic acid.^24^ At 30 µmol·L arachidonic acid, ACT’s inhibitory action was reduced, only COX3 was inhibited with an IC_50_ value of 460 µmol·L.^24^ Here however, it is important to clarify that cells containing COX1 and COX2 produced more PGE2 than cells containing COX3 in the absence of ACT (COX1 containing cells 5‐fold and COX2 containing cells 25‐fold greater PGE2 production than COX3 containing cells^24^, ^31^). Therefore, the apparent potency of ACT on COX3 may be a consequence of the low rate of PGE2 production by COX3.^31^, ^32^ With only one study completing this type of analysis^24^ further assessment of COX1‐3 sensitivity to ACT is required.^31^

Since the discovery of the COX3 enzyme and its apparent sensitivity to ACT^24^ research has sought to determine if this is how ACT exerts its action, positing that this explains why ACT does not display anti‐inflammatory and antiplatelet function.^20^, ^24^, ^25^, ^26^ To investigate this, COX3 hypothesis studies have assessed the analgesic (acetic acid/iloprost induced writhing in mice) and antipyretic/hypothermic (i.e., PGE2 inhibition) function of ACT.^20^, ^25^, ^26^, ^35^, ^36^ The writhing responses to acetic acid or iloprost injection were dose dependently reduced by ACT; however, diclofenac (a non‐selective NSAID) only reduced acetic acid‐induced writhing.^26^ Iloprost‐induced writhing is not reduced by peripherally acting drugs like NSAIDS; the anti‐inflammatory or antiplatelet ability of NSAIDS is generally a result of peripheral COX inhibition.^26^, ^37^ This exhibits ACT’s greater central mechanism of action^26^, ^31^ with the authors citing COX3, only observed in the brain [i.e., centrally^24^], as the target of the analgesic effects of ACT in these mice.^26^ ACT is not only antipyretic but hypothermic [i.e., reduces afebrile core temperature (Tc)] in rodents and humans.^25^, ^35^, ^36^ ACT‐induced afebrile Tc reduction appears to be a direct result of PGE2 inhibition in mice^25^ (a mechanism also hypothesized in humans but yet to be confirmed^38^, ^39^). More recently, COX3 inhibition has been extended to ACT’s febrile Tc reduction,^20^ in contrast to previous research which observed COX3 to be unresponsive to acute inflammation.^40^ The authors cite the loss of potent hypothermic and antipyretic action in COX1 knockout mice^20^, ^25^ and the fact that COX1 selective and dual COX1/2 inhibitors failed to induce afebrile hypothermia^20^ as evidence of COX3 inhibition by ACT. However, the use of COX1 knockout mice to assess the function of this COX3 enzyme may not be experimentally sound, as in COX1 knockout mice, gene targeting disrupts the C terminal of COX1.^41^ Any protein derived (e.g., COX3) from this would be without the 120 C terminal acids central to the enzymatic activity of COX1,^41^, ^42^ but (and importantly), would contain the entire sequence for the COX3 protein.^41^ It is improbable, therefore, that COX3 would be involved in prostaglandin synthesis for pain and/or thermoregulation.^41^ Indeed, evidence for COX3 as the target of ACT’s action is far from unequivocal. In similar experiments COX3 was not found to be involved in either the antipyretic or hypothermic action of ACT.^27^ Additionally, one of the key arguments for COX3 being the target of ACT is that the drugs aminopyrine and antipyrine, apparent COX3 selective inhibitors, elicit similar analgesic and antipyretic/hypothermic responses as ACT in mice.^20^, ^25^, ^26^ Importantly, the premise for these drugs being selective COX3 inhibitors is from the same study that first identified the existence of this COX3 enzyme and the potential for it to be a target for ACT.^24^ Much like ACT, these drugs (aminopyrine and antipyrine) are considered to be mild analgesics with weak inhibition of the well‐recognized COX1/2 isoforms with their precise mechanism of action still debated.^43^, ^44^

The work described here mainly details results from mammalian species other than humans despite the focus of this paper is human in vivo physiology. Namely, human ACT/COX3 data are not available. It is plausible that undiscovered COX isoforms and splice variants could display germane physiological functions^22^ nevertheless, the current evidence for COX3 as the elusive target of ACT are inconclusive:

1. COX3 protein has been detected in human tissues^23^ but no functional COX3 enzyme has been sequenced.^20^, ^32^ Multiple COX variants have been sequenced in rodent and human models; however, no physiological or pathological functions have been ascribed to these variants and there is no evidence that they are a target for ACT.^22^, ^28^ Indeed, ~50% of human genes may produce mRNA products that are unproductive targets for degradation.^45^ COX3, a splice variant of COX1, may be an example of one of these products.
2. The proposed evidence of COX3 as a target of ACT may not be experimentally sound (i.e., use of COX1 knockout mice to model COX3 activity/function^41^ and the interpretation of COX3 sensitivity to ACT may be a direct consequence of low catalytic activity not inhibition^31^, ^32^).
3. Translation of data from other mammalian species is often inappropriate due to large interspecies differences. There are vast differences across mammalian species (e.g., body size and hair coverage) that make the translation to humans challenging.^28^, ^46^ Furthermore, even between rodent species and different strains of the same species there are differences in the response to ACT administration.^47^ Indeed, the COX3 enzyme shown to exhibit COX activity in mice^24^ has been cloned in rats but does not exhibit COX activity.^29^

Based on the current evidence, we hypothesize that ACT does not act on COX3 (in acceptance of hypothesis i).

## 3 COX2 SELECTIVITY OF ACETAMINOPHEN

To the authors’ knowledge, there are two human studies that provide evidence in support of ACT as a selective COX2 inhibitor.^30^, ^48^ In vitro, ACT displayed a 4.4‐fold selectivity for COX2 and in vivo ACT average plasma concentrations were below the IC_50_ value for COX1 but greater than or equal to the IC_50_ value for COX2.^30^ Ex vivo concentrations of thromboxane B2 [TXB2 (COX1 pathway)] and lipopolysaccharide (LPS) induced PGE2 (COX2 pathway) represented an 83% inhibition of COX2 compared to 56% COX1.^30^ This level of COX2 inhibition is similar to that of other selective COX2 inhibitors^49^; however, the 4.4‐fold selectivity for COX2 over COX1 is considerably lower than that observed in other selective COX2 inhibitors (30–433 fold greater inhibition of COX2 than COX1^50^). Furthermore, other COX2 selective inhibitors do not exhibit such high COX1 inhibition; etoricoxib and celecoxib inhibited ex vivo TXB2 (i.e., COX1) by 15.5% (95% CI: 6.6 – 23.5) and 20.2% (95% CI: 11.5–28.1), respectively.^51^ The conclusion that the greater COX2 inhibition by ACT demonstrates COX2 selectivity^30^ is therefore somewhat questionable.

In response to a clinical model of inflammation, ACT only suppressed in vivo PGE2 (and not TXB2) similar to rofecoxib (a selective COX2 inhibitor) suggesting that in vivo ACT selectively inhibits COX2.^48^ It is important to note the removal of two impacted third molars induces a pathophysiological state where COX2 (inducible in response to pathology) is likely the predominant functioning COX isoform.^12^ Similarly, in rodent models where a pathophysiological state (i.e., fever) is induced via LPS injection it is ACT COX2 inhibition that prevents and/or reduces high Tc resulting from LPS induced fever.^27^, ^52^ The greater COX2 inhibition exhibited in these studies^30^, ^48^ is not conclusive evidence that ACT is selective in its COX inhibition. Indeed, in vitro ACT can be a potent inhibitor of COX1 when peroxide concentrations are low, although supratherapeutic concentrations were used.^53^

**FIGURE 2:**
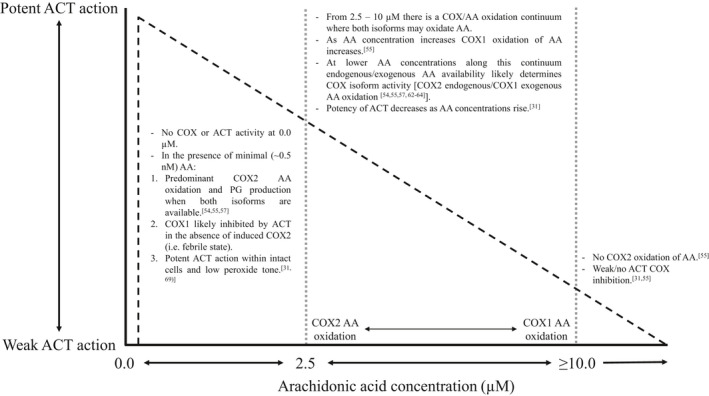
Schematic of hypothesis 2: ACT is not selective in its COX inhibition and hypothesis 3: ACT inhibition of COX isoforms is determined by subtle and nuanced physiological variations. A graphical representation of how COX activity and arachidonic acid concentration interplay to determine the isoform ACT inhibits and the potency with which ACT inhibits this isoform. Abbreviations: AA, arachidonic acid; ACT, acetaminophen; COX, cyclooxygenase

When attempting to ascribe COX selectivity to ACT it is imperative to understand the biological conditions that determine COX activity. COX1 and COX2 oxidation of arachidonic acid occurs under separate conditions and has been termed the arachidonic acid rule.^54^ It appears that COX1 can utilize concentrations of arachidonic acid >10 µM, concentrations of this magnitude only occur when arachidonic acid is exogenously increased in the cell whereas, concentrations ≤2.5 µM are released endogenously and COX2 has 2‐ to 4‐fold greater activity than COX1.^55^, ^56^, ^57^ At arachidonic acid concentrations between 50 nM and 1 µM, COX1 produces less than 25% of the “product” of COX2.^57^ Importantly, this concentration range is likely what is available in vivo.^58^ Arachidonic acid is subject to a reacylation/deacylation cycle that keeps concentrations very low^59^, ^60^, likely to avoid cytotoxicity that can occur if concentrations exceed 50–100 µM in vitro^59^ (human plasma arachidonic acid concentrations can reach 500 µM^59^, ^61^). There are not precise concentrations of arachidonic acid that determine oxidation by COX1 or COX2.^55^, ^56^, ^57^, ^62^, ^63^, ^64^ Between 2.5 and 10 µM, COX1 shows greater activity in arachidonic acid oxidation than COX2,^57^ this is likely a result of COX1 requiring cooperative activation (higher substrate concentration, i.e., arachidonic acid) while COX2 does not.^57^ Perhaps more conceivably it was the specific biological in/ex vivo conditions (i.e., fever/inflammation^30^, ^48^) alongside a low sample size in vitro and in vivo (*n* = 5^30^) that accounted for the observed greater inhibition of COX2. In the immediate response to pathological stimuli (i.e., inflammation/fever), there is an intense activation of phospholipases that release a burst of arachidonic acid beyond the threshold of COX2 utilization.^54^ Therefore, COX1 may provide the immediate febrile response.^54^ In this initial stage of the febrile response, the ability of ACT to exert its action is likely to be diminished due to high peroxide tone.^31^, ^53^ However, as arachidonic acid concentrations fall below the threshold of COX1 oxidation, COX2 becomes the isoform responsible for the febrile response^54^ and ACT potently exerts its action.^31^ Under these conditions of low concentrations of arachidonic acid the COX2 pathway is preferred to COX1^64^—therefore—it may seem, albeit potentially incorrectly, as if ACT is selectively inhibiting COX2 (Figure 2 depicts the COX/arachidonic acid relationship and ACT selectivity).

As discussed previously ACT can induce a hypothermic effect in afebrile mammals.^25^, ^35^, ^36^ Febrile increases in Tc result from PGE2 (the pyrogenic mediator^65^) upregulation from inducible COX2, inhibition of COX2 (e.g., ACT/NSAID) prevents PGE2 synthesis and reduces fever.^66^ Evidence of COX2 constitutive expression/function is limited^14^, ^15^, ^16^, ^17^ and it is unlikely that inhibition of COX2 derived PGE2 is responsible for the reductions in afebrile Tc following ACT administration observed in mice^25^ and humans.^35^, ^36^ This hypothermic effect occurs in mammals housed below their thermoneutral zone,^25^, ^35^, ^36^ conditions that require heat generation (i.e., thermogenesis) to maintain homeostatic Tc.^67^ In afebrile mice, ACT‐induced Tc reductions were simultaneous with 96% reductions in PGE2^25^ suggesting that PGE2 may be involved in afebrile Tc regulation. Evidence of ACT inhibitory action on COX3 is equivocal and robustly refuted (discussed above); nevertheless, the data suggest inhibition of COX1 (or COX1‐derived isoform) not COX2 is responsible for ACT induced afebrile Tc reductions.^20^, ^25^, ^26^ In vivo analysis (i.e., COX/PGE2 concentrations) is required in humans to determine whether ACT induced COX1 inhibition is the cause of this afebrile hypothermic effect.

In vivo biological variations determine COX activity (i.e., COX1/2 derived PG’s) and this appears to directly affect the potency of ACT and the COX isoform it inhibits; ACT can appear to be COX2 selective but current evidence does not support this notion.^31^ Based on the work described here we hypothesize ACT is not selective in its COX inhibition (in acceptance of hypothesis ii) but subtle in vivo biological variations dictate the COX isoform inhibited by ACT (in acceptance of hypothesis iii).

## 4 PHYSIOLOGICAL VARIATIONS DICTATE ACETAMINOPHEN COX INHIBITION

Arachidonic acid concentrations in vivo (i.e., physiological conditions) determine which COX isoform PG’s are derived from and subsequently influences the isoform ACT inhibits.^31^ ACT’s efficacy is increased when arachidonic acid concentrations are low, which are generally concomitant with low peroxide tone within cells.^31^ Figure 2 represents the relationship between arachidonic acid and ACT potency. More potent COX1 inhibition by ACT occurs at low peroxide concentrations,^53^ and more recently, this has been extended to intact cells.^68^ Broken cells and/or exogenous increases in intracellular peroxide tone in intact cells abolish the COX inhibitory effects of ACT in vitro.^68^, ^69^ Within intact cells ACT COX1 inhibition is evidenced to occur when exogenously added arachidonic acid concentration is low or in the presence of cytokines (e.g., interleukin 1β) that release arachidonic acid in low concentrations.^69^ ACT’s efficacy is higher under these conditions because low arachidonic acid concentrations result in low PGG2 (a hydroperoxide) within cells.^31^ As described, independent COX2 oxidation of arachidonic acid occurs at lower concentrations (≤2.5 µM) than independent COX1 oxidation (>10 µM^64^), giving the perception that ACT is COX2 selective and accounts for its lack of anti‐inflammatory and antiplatelet activity where high concentrations of peroxides are present.^31^, ^69^ Concentrations of arachidonic acid at COX1 oxidation levels (>2.5 µM) are still considered low (i.e., not cytotoxic^59^) therefore, assuming that COX3 is not the target of ACT, the loss of hypothermic and analgesic properties in COX1 knockout mice, but not COX2 knockout mice,^25^, ^26^ may evidence COX1 inhibition by ACT under low arachidonic acid concentration/peroxide tone conditions^31^; however, specific human in vivo data is required to confirm this assertion.

Illustrated here is the intricacy of determining the specific in vivo action of a drug and the activity of complex molecules/biomarkers in mammalian species.^18^, ^19^ Much of the data presented here requires confirmation from human in vivo research. However, we maintain that on current evidence it is the subtle in vivo biological variations that determine the COX isoform inhibited by ACT (in acceptance of hypothesis iii), ACT is not a selective COX2 inhibitor (in acceptance of hypothesis ii) and COX3 is not the target of ACT inhibition (in acceptance of hypothesis i).

## 5 NON‐COX‐RELATED MECHANISMS OF ACTION

The main focus of this article is the COX–ACT‐related mechanism of action; however, it is important to acknowledge recent evidence that suggests the analgesic effects exhibited by ACT may be a result of action via non‐COX‐related pathways [for a more in‐depth review see Ohashi and Kohno^70^]. In brief, transient receptor potential vanilloid 1 (TRPV1) and cannabinoid 1 (CB1) receptors are involved in pain modulation.^71^, ^72^ TRPV1 in the dorsal raphe nucleus^71^ and both TRPV1 and CB1 in the rostral ventromedial medulla.^72^ Activation of the TRPV1/CB1 receptors in these regions induce analgesia.^70^ ACT is metabolized to *p*‐aminophenol that is converted to N‐acylphenolamine (AM404) once it crosses the blood‐brain barrier.^73^ AM404 is known to act on TRPV1 and CB1 receptors,^73^ action that has recently been observed to produce analgesia.^70^, ^74^ This ACT, AM404 and TRPV1/CB1 receptor pathway appears to have a significant role in the analgesic effects of ACT and proffers an explanation to its central prolonged mechanism of action.^70^ In addition, ACT has been cited to activate the serotonergic inhibitory pathway, a pathway also known to be important in the modulation of pain.^75^ Inhibition of serotonergic receptors (those implicated: serotonin [5‐HT]_1A_, 5‐HT_3,_ and 5‐HT_7_) has been shown to eradicate any analgesic action of ACT^76^, ^77^, ^78^, ^79^, ^80^, ^81^, ^82^, ^83^ and reductions in serotonin levels reduces the analgesic efficacy of ACT.^84^ ACT‐induced activation of this pathway does not, however, elucidate the analgesic mechanism of action as it has been shown that ACT lacks any affinity to serotonergic receptors.^85^ How ACT interacts with this serotonergic pathway is not yet confirmed. Further research exploring the nuances of COX and non‐COX‐related ACT mechanisms of action are evidently required. Such data may also shed light on the COX‐PGE2‐ACT mechanisms of actions discussed above.

## 6 RECOMMENDATIONS FOR FUTURE RESEARCH

The hypotheses of this paper require further exploration. Despite decades of research, the precise mechanism of action and/or pharmacological target of ACT is still not fully understood.^20^, ^21^ We hypothesize that COX3 is not the target of ACT’s action; however, it is possible that an unidentified COX isoform or splice variant may be the target of ACT.^22^ Identification of the pharmacological target of ACT represents the possibility of alternative methods to pharmacologically treat pain and/or fever.^20^, ^21^ Specific in vivo human research is required, due to the discussed issues with translation of data from rodents to humans.^28^, ^46^ The evidence available does not support the notion that ACT selectively inhibits COX2; however, it may predominantly inhibit COX2 based on the subtle in vivo biological conditions (i.e., arachidonic acid/peroxide tone concentrations) that favor ACT COX2 inhibition. Much of the research presented focuses on acute doses of ACT to determine mechanism of action. Understanding the nuances of chronic ACT use and COX inhibition is a prevalent research question. Prolonged COX2 inhibition poses a cardiovascular risk, chronic use of NSAIDs, and selective COX2 inhibitors have exhibited this side effect^86^ leading to the withdrawal of rofecoxib (COX2 selective) from the market.^87^ The risk of cardiovascular adverse events from ACT use is debated^88^; however, there is some evidence of a dose‐response relationship with increased cardiovascular adverse events.^89^ Further investigation is required to elucidate potential ACT cardiovascular risk. This paper presents strong evidence that the COX isoform inhibited by ACT is dependent on subtle biological variations in vivo. Given there is no definitive consensus of how ACT induced COX inhibition occurs, further research is required specifically focusing on the biological conditions that may alter ACT efficacy and COX inhibition.

## 7 CONCLUSION

Despite being in use as early as the 1890s (more commonly from the 1950s) and becoming one of the most prevalently used analgesic/antipyretic drugs worldwide the specific mechanism of action of ACT is not fully elucidated. Research attempting to discern its mechanism of action have been collated within this paper and based on current work this paper accepts the hypotheses that ACT: (i) does not act on a third COX isoform; (ii) is not selective in its COX inhibition; and (iii) inhibition of COX isoforms is determined by subtle and nuanced biological variations. Importantly, there is a need for further robust research designs to confirm these hypotheses conclusively.

## 8 NOMENCLATURE OF TARGETS AND LIGANDS

Key protein targets and ligands in this article are hyperlinked to corresponding entries in http://www.guidetopharmacology.org, the common portal for data from the IUPHAR/BPS Guide to PHARMACOLOGY,^90^ and are permanently archived in the Concise Guide to PHARMACOLOGY 2019/20.^91^

## DISCLOSURE

The authors have no conflicts of interest.

## CONSENT STATEMENT/ETHICAL APPROVAL

Not required.

## Data Availability

Data sharing is not applicable to this article as no new data were created or analyzed in this study.
